# Proteomic Analysis of Biomarkers Predicting Treatment Response in Patients with Head and Neck Cancers

**DOI:** 10.3390/ijms252312513

**Published:** 2024-11-21

**Authors:** Emeshaw Damtew Zebene, Rita Lombardi, Biagio Pucci, Hagos Tesfay Medhin, Edom Seife, Elena Di Gennaro, Alfredo Budillon, Gurja Belay Woldemichael

**Affiliations:** 1 Nuclear Medicine Unit, Department of Internal Medicine, College of Health Sciences, Addis Ababa University, Addis Ababa 9086, Ethiopia; emeshaw.damtew@aau.edu.et (E.D.Z.); hagos.tesfay@aau.edu.et (H.T.M.); 2 Department of Microbial Cellular and Molecular Biology, College of Natural and Computational Sciences, Addis Ababa University, Addis Ababa 9086, Ethiopia; gurja.belay@aau.edu.et; 3 Experimental Animal Unit, Istituto Nazionale Tumori-IRCCS-Fondazione G. Pascale, 80131 Naples, Italy; r.lombardi@istitutotumori.na.it; 4 Experimental Pharmacology Unit-Laboratory of Naples and Mercogliano (AV), Istituto Nazionale Tumori-IRCCS-Fondazione G. Pascale, 80131 Naples, Italy; e.digennaro@istitutotumori.na.it; 5 Radiotherapy Center, College of Health Sciences, Addis Ababa University, Addis Ababa 9086, Ethiopia; edomseife3@gmail.com; 6 Scientific Directorate, Istituto Nazionale Tumori-IRCCS-Fondazione G. Pascale, 80131 Naples, Italy; a.budillon@istitutotumori.na.it

**Keywords:** biomarker, head and neck cancer, proteomics, chemotherapy, radiotherapy

## Abstract

Head and neck cancers (HNCs) are the sixth most commonly diagnosed cancer and the eighth leading cause of cancer-related mortality worldwide, with squamous cell carcinoma being the most prevalent type. The global incidence of HNCs is steadily increasing, projected to rise by approximately 30% per year by 2030, a trend observed in both developed and undeveloped countries. This study involved serum proteomic profiling to identify predictive clinical biomarkers in cancer patients undergoing chemoradiotherapy (CRT). Fifteen HNC patients at Tikur Anbessa Specialized Hospital, Radiotherapy (RT) center in Addis Ababa were enrolled. Serum samples were collected before and after RT, and patients were classified as responders (R) or non-responders (NR). Protein concentrations in the serum were determined using the Bradford assay, followed by nano-HPLC–MS/MS for protein profiling. Progenesis QI for proteomics identified 55 differentially expressed proteins (DEPs) between R and NR, with a significance of *p* < 0.05 and a fold-change (FC) ≥ 1.5. The top five-up-regulated proteins included *MAD1L1*, *PSMC2*, *TRIM29*, *C5*, and *SERPING1*, while the top five-down-regulated proteins were *RYR1*, *HEY2*, *HIF1A*, *TF*, and *CNN3*. Notably, about 16.4% of the DEPs were involved in cellular responses to DNA damage from cancer treatments, encompassing proteins related to deoxyribonucleic acid (DNA) damage sensing, checkpoint activation, DNA repair, and apoptosis/cell cycle regulation. The analysis of the relative abundance of ten proteins with high confidence scores identified three DEPs: *ADIPOQ*, *HEY2*, and *FUT10* as potential predictive biomarkers for treatment response. This study highlighted the identification of three potential predictive biomarkers—*ADIPOQ*, *HEY2*, and *FUT10*—through serum proteomic profiling in HNC patients undergoing RT, emphasizing their significance in predicting treatment response.

## 1. Introduction

Cancer is likely to emerge as a leading cause of death and a significant obstacle to global life expectancy, currently ranking as the second leading cause of death globally [1]. Specifically, head and neck cancers (HNCs) are the sixth most commonly diagnosed cancer and the eighth leading cause of cancer-related mortality worldwide, with squamous cell carcinoma being the most prevalent type [2]. The global incidence of HNC is steadily increasing, projected to rise by approximately 30% annually by 2030, a trend observed in both developed and developing countries [3].

Head and neck squamous cell carcinoma (HNSCC) refers to a diverse range of cancers that develop in the epithelial cells of mucosal linings found in various anatomical locations, such as the nasopharynx, paranasal sinuses, oral cavity, oropharynx, hypopharynx and larynx. These malignancies are primarily linked to the consistent use of tobacco and alcohol, as well as infection with high-risk strains of the human papilloma virus (HPV) infection. The risk of developing HNSCC is 2–4 fold higher in men than in women [4,5].

According to global cancer observatory (GLOBOCAN) estimates, HNSCC represents approximately 4.5% of cancer diagnoses and fatalities, with around 890,000 new cases and 450,000 deaths occurring each year. In Africa, the prevalence of cancer-related incidence and deaths are 5.7% and 7.2%, respectively [5]. Among the continent’s 54 countries, only 19 have conducted research on HNSCC, with Nigeria leading at 27%, followed by South Africa and Tunisia at 12% each [6].

Surgery, chemotherapy, and radiotherapy either alone or in combination are the standard treatment options for HNSCC. Even in the presence of better treatment preferences, HNSCC still has a poor prognosis, with an overall survival rate of nearly 65%. The outcome of managing patients with HNSCC is influenced by various factors, including the natural progression of the disease, tumor biology, and stage of the cancer. Additionally, the stratification of therapy by assessing the overall condition of the patient and making clinical observations of the tumor using the location and size of the tumor do not have a significant impact on patient prognosis [7,8]. These elements collectively affect the course of treatment and ultimately determine the patient’s overall outcome. Clinical observation of the tumor refers to the systematic monitoring and assessment of a tumor’s characteristics, such as its size, location, and any changes over time, without immediate intervention or treatment. HNCs methods for stratifying therapy—specifically through assessing the overall condition of the patient and observing tumor characteristics like its location and size—may not significantly influence patient outcomes. This could be due to different factors such as the tumor’s biology [9,10], in which the inherent aggressiveness of the tumor may play a more critical role in prognosis than external factors like its size or location. Additionally, in advanced stages of the disease, factors such as metastasis might outweigh the importance of tumor characteristics in determining prognosis [11].

Over the last decade, technological advancements have significantly enhanced our understanding of the molecular mechanisms underlying neoplastic transformation and disease progression in HNSCC. Numerous biomarkers have been proposed, but their successful translation into clinical practice remains limited [12]. Despite large efforts, HNSCC remains a tumor type that lacks personalized treatment options. As a result, the primary approaches for treating HNSCC in curative cases continue to be surgery and radiotherapy, sometimes combined with systemic therapy, including immunotherapy, which has emerged as a promising treatment option, particularly for recurrent or metastatic HNCs [7]. Unlike other cancers, where molecular profiling is commonly used to guide the choice between more aggressive or less aggressive treatment approaches [13,14], the treatment of HNSCC lacks such molecular stratification. To address this limitation, there is a pressing need to develop biomarkers that could facilitate the early detection of treatment responses, enable personalized therapy approaches, and ultimately lead to better patient outcomes while optimizing healthcare resources [12,15].

Additionally, developing supportive care interventions for HNC survivors is crucial, as these patients often experience significant treatment-related side effects that impact their quality of life [16]. Strategies for evidence-based practice in HNC care should incorporate systematic reviews to guide clinical decision making and improve supportive care [17]. Although transcriptomic and DNA methylation profiling have been successful in identifying cancer biomarkers, relying solely on DNA or Ribonucleic acid (RNA) information is insufficient for determining the optimal cancer drugs, as most cancer therapies are designed to target specific proteins [18]. It is often challenging to directly correlate a genetic mutation with the anticipated protein alteration. Consequently, proteomics has emerged as a highly promising tool for discovering biomarkers in recent years [19]. The introduction of mass spectrometry (MS)-based protein analysis technology has enabled the widespread adoption of large-scale protein analysis. Nanoscale liquid chromatography combined with tandem mass spectrometry (nano LC–MS/MS) has become a fundamental tool in proteomics [20].

In this study, we analyzed serum samples from HNC patients who received chemoradiotherapy (CRT), aiming to use nano LC–MS/MS-based serum proteomics to identify clinical predictive biomarkers of treatment response.

## 2. Results

### 2.1. Proteome Profiles of Serum Samples from Head and Neck Cancer Patients

**Figure 1:**
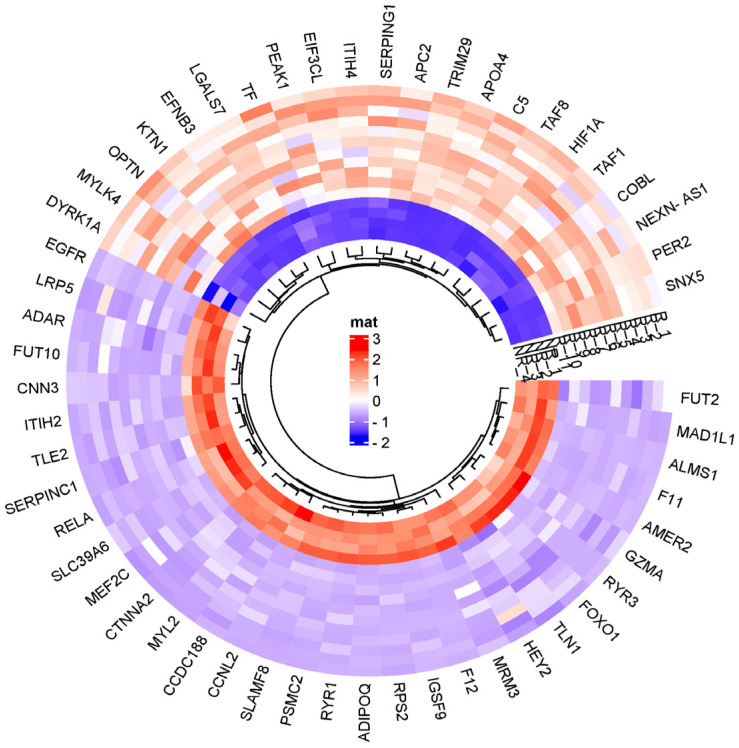
Hierarchical cluster of DEPs in both Responders and Non-Responders with a fold-change ≥ 1.5 and *p*-value <0.05. The expression level is indicated by the intensity of the color: red, high expression; blue, low expression.

Label-free quantitative proteomics was used to identify and quantify proteins that exhibited significantly different abundances before and after treatment between responder (R) and non-responder (NR) groups. Using the Progenesis QI software, 55 differentially expressed proteins (DEPs) were identified based on the following criteria: FC ≥ 1.5 and ANOVA test *p*-value < 0.05 (Supplementary Table S1). Among these, 22 were up-regulated and 33 were down-regulated (Figure 1). The FCs were separately calculated for both the R and NR groups with respect to their baseline and presented in Table 1. These DEPs were generally discussed in terms of their regulations (up/down) and their functional interactions, such as their molecular functions, biological processes, and/or pathways.

Of the 55 DEPs identified, the top five up-regulated proteins were the Mitotic Spindle Assembly Checkpoint Protein MAD1 (*MAD1L1*), 26S proteasome regulatory subunit 7 (*PSMC2*), Tripartite motif-containing protein 29 (*TRIM29*), complement C5 (*C5*), and Plasma protease C1 inhibitor (*SERPING1*). The top five down-regulated proteins were the Ryanodine Receptor 1 (*RYR1*), Hairy/enhancer-of-split related with YRPW motif protein 2 (*HEY2*), Hypoxia-inducible factor 1-alpha (*HIF1A*), Serotransferrin (*TF*), and Calponin-3 (*CNN3*).

**Figure 2:**
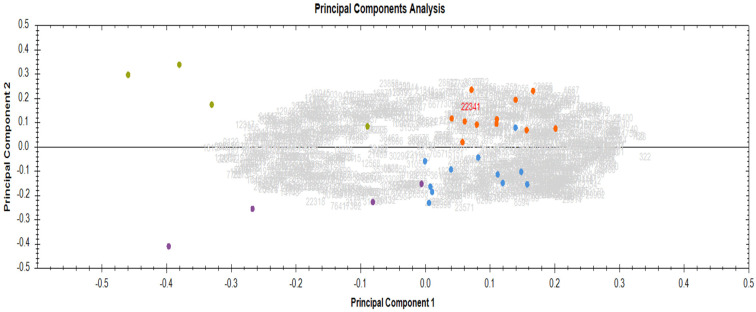
Principal component analysis highlighting the close clustering of biological replicates between Responders and Non-Responders (baseline vs end of radiotherapy). Baseline R (blue), baseline NR (purple), End RT_R (orange), End RT_NR (green).

Principal component analysis (PCA), derived from an unsupervised multivariate analysis of DEPs, demonstrated the good experimental reproducibility of the serum proteomics results, as indicated by the close clustering of biological replicates into four groups: baseline R (blue), baseline NR (purple), End RT_R (orange), and End RT_NR (green) (Figure 2).

### 2.2. Gene Ontology (GO)Analysis

**Figure 3:**
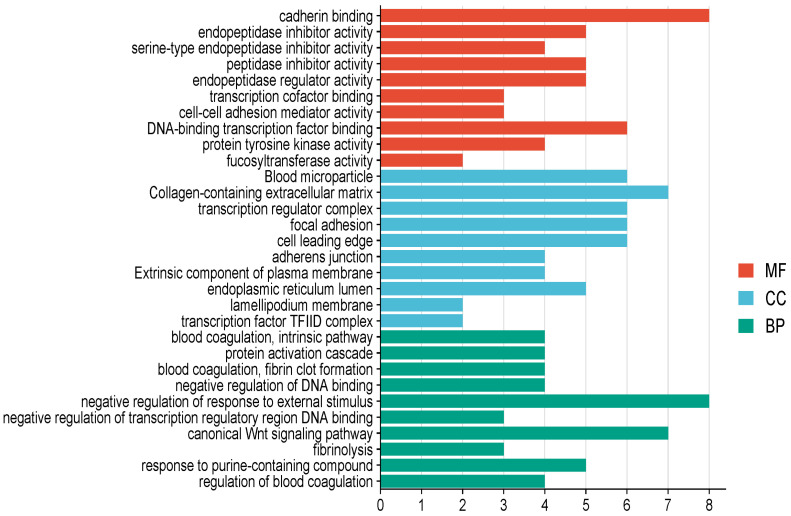
Gene ontology classification analysis of DEPs. The number of DEPs in the three ontology classifications: Molecular function (MF), Cellular component (CC), Biological process (BP). The horizontal axis represents the number of overlapping proteins, while the vertical axis represents the ontology classification name.

GO classification was performed to analyze the function of differentially expressed proteins according to three terms: biological process (BP), molecular function (MF), and cellular components (CC). Figure 3 shows the gene ontology classification analysis of the DEPs. Briefly, in BP classification, negative regulation of responses to external stimuli, the canonical Wnt signaling pathway, responses to purine-containing compounds, negative regulation of DNA binding, and different aspects of blood coagulation pathways were the major functional classes of the DEPs. Twelve (22%) of the 55 DEPs were involved in the immune and metabolism of biological processes. These include *TRIM29*, Transcription initiation factor TFIID subunit 8 (*SLAMF8*), *C5*, *SERPING1*, *TF*, Granzyme A (*GZMA*), Transcription factor p65 (*RELA*), Apolipoprotein A-IV (*APOA4*), Adiponectin (*ADIPOQ*), Galactoside alpha-(1,2)-fucosyltransferase 2 (*FUT2*), 40S ribosomal protein S2 (*RPS2*), and Adenomatous polyposis coli protein 2 (*APC2*). In the MF classification, cadherin binding, DNA-binding transcription factor binding, endopeptidase inhibitor activity, peptidase inhibitor activity, and endopeptidase regulator activity were the major functional classes. In CC classification, collagen-containing extracellular matrix, blood microtubules, transcription regulator complexes, focal adhesion, and cell leading age were the major components of the DEPs.

### 2.3. Pathway Analysis

**Figure 4:**
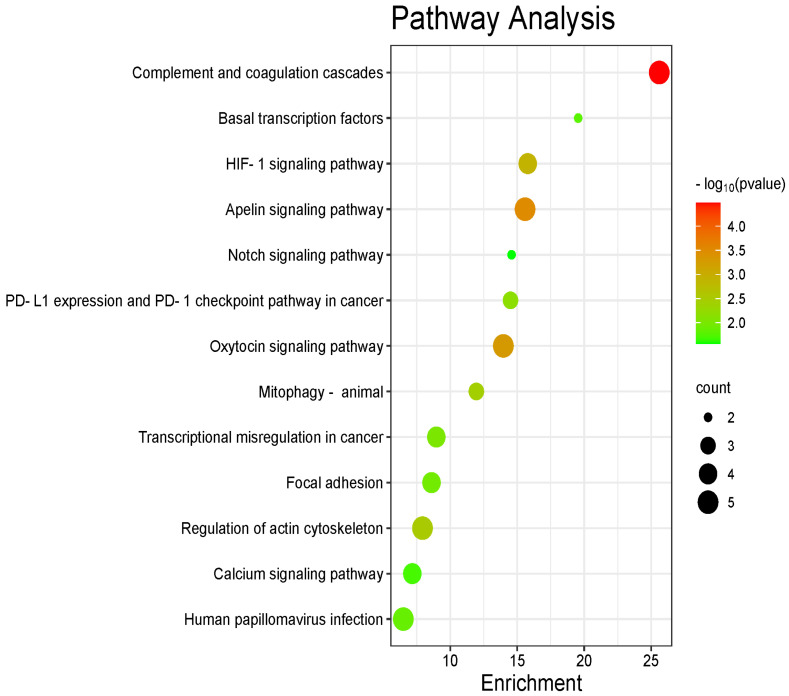
Dot plot of the KEGG pathway enrichment analysis. The horizontal axis represents the DEP ratio, while the vertical axis represents the enrichment pathway name. The color scale indicates different thresholds of the *p*-value, and the size of the dot indicates the number of DEPs corresponding to each pathway.

In order to see the role of these DEPs, we performed a KEGG pathway analysis, and 13 KEGG pathways were significantly enriched based on proteins with a *p*-value < 0.05 (Figure 4). The enriched KEGG pathways are provided in Supplementary Table S2. The top ten enriched pathways are the complement and coagulation cascades, Apelin signaling pathway, oxytocin signaling pathway, *HIF-1* signaling pathway, regulation of actin cytoskeleton, mitophagy-animal, PD-L1 expression and PD-1 checkpoint pathway, transcriptional dysregulation in cancer, focal adhesion, and human papilloma virus infection (HPV).

**Figure 5:**
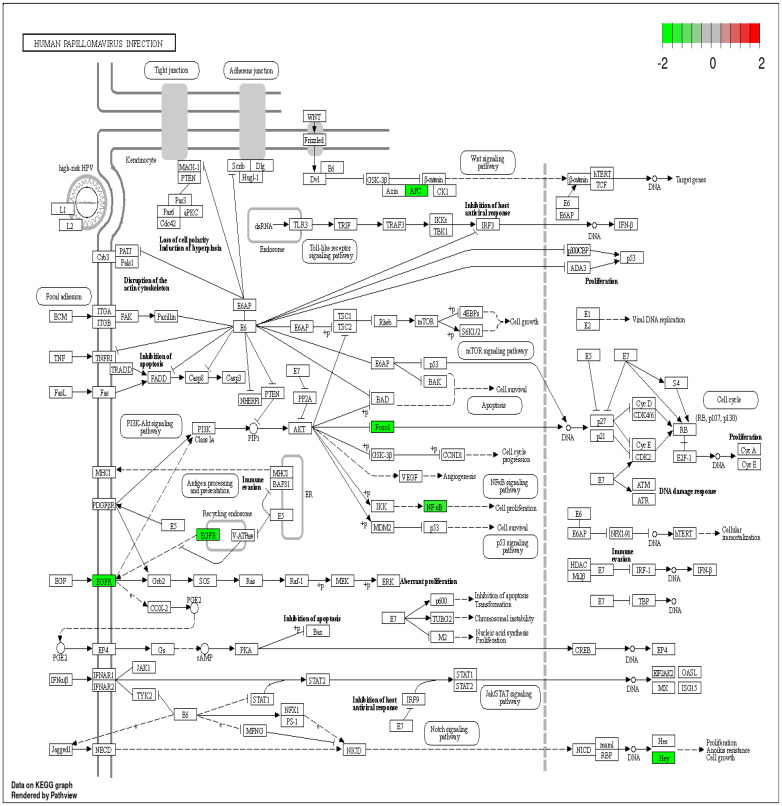
Human papilloma virus infection pathway with differentially expressed proteins (DEPs). The down-regulated DEPs are highlighted in a green color.

DEPs such as the Forkhead box protein O1 (*FOXO1*), epidermal growth factor receptor (*EGFR*), *RELA*, APC2, and *HEY2* were involved in HPV infection. A significant subset of carcinomas developed in HNCs (40–60% of oropharyngeal cancers) are associated with specific High-risk human papilloma virus (HR-HPV) genotypes [21]. Furthermore, signaling pathways in HR-HPV-driven head and neck cancers have been well characterized [22]. Therefore, DEPs enriched in this pathway (Figure 5) may play an important role in HNCs.

**Figure 6:**
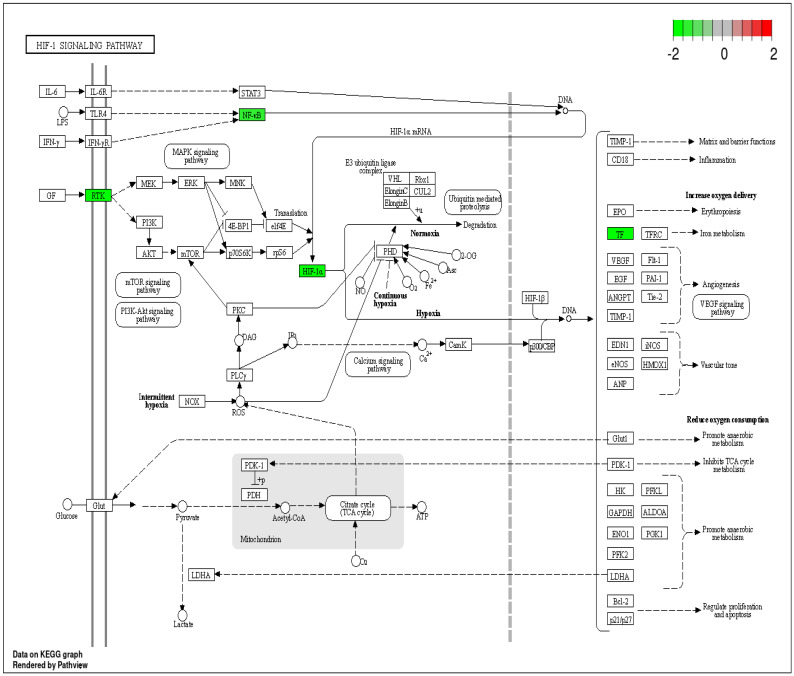
HIF-1 signaling pathway with differentially expressed proteins (DEPs). The down-regulated DEPs are highlighted in a green color.

Additionally, DEPs such as RELA, HIF-1α, and TF were involved in the HIF-1 signaling pathway. HIF-1α is an oxygen-inducible factor that is implicated in various physiological and pathological conditions, including cancers such as HNCs, where it promotes tumor growth, angiogenesis, and resistance to chemo and radiotherapy. Moreover, many HIF-1 inhibitors have been developed and evaluated as potential anticancer drugs in pre-clinical and clinical studies [23]. Hence, the DEPs enriched in this pathway (Figure 6) may play a great role as prognostic predictors of HNCs.

**Figure 7:**
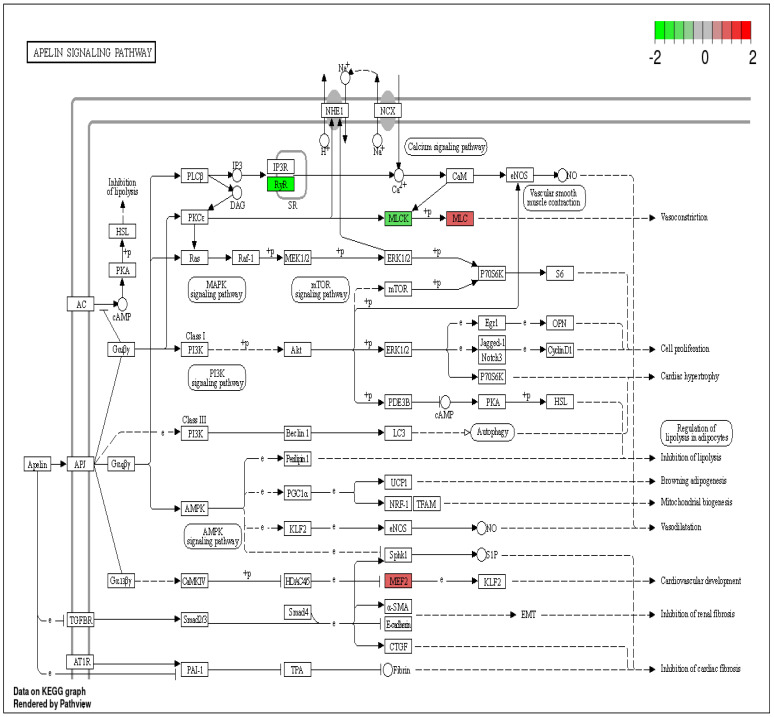
Apelin signaling pathway with differentially expressed proteins (DEPs). The up-regulated DEPs are highlighted in a red color and the down-regulated DEPS are highlighted in a green color.

Furthermore, DEPs such as *RyR*, *MYLK4*, Myosin regulatory light chain 2, ventricular/cardiac muscle isoform (*MYL2*), and Myocyte-specific enhancer factor 2C (*MEF2C*) are implicated in the apelin signaling pathway. Recent studies have indicated that the apelin (APL) signaling pathway is involved in various physiological and pathological processes, including cancer [24]. In cancer cells, apelin signaling has been shown to promote tumor growth through mechanisms involving the PI3K/Akt pathway, as well as the activation of Notch3 and STAT3 [25]. Notably, apelin expression has been significantly linked to tumor recurrence and disease-free survival in oral squamous cell carcinoma (OSCC). Under hypoxic conditions, apelin expression increases, and the addition of exogenous apelin has been found to enhance the proliferation and migration of the oral cancer cell line HSC-3 by promoting the phosphorylation of *ERK1/2* [26]. Therefore, the DEPs that are enriched in the apelin signaling pathway (Figure 7) may play a crucial role in the mechanisms underlying HNCs.

**Figure 8:**
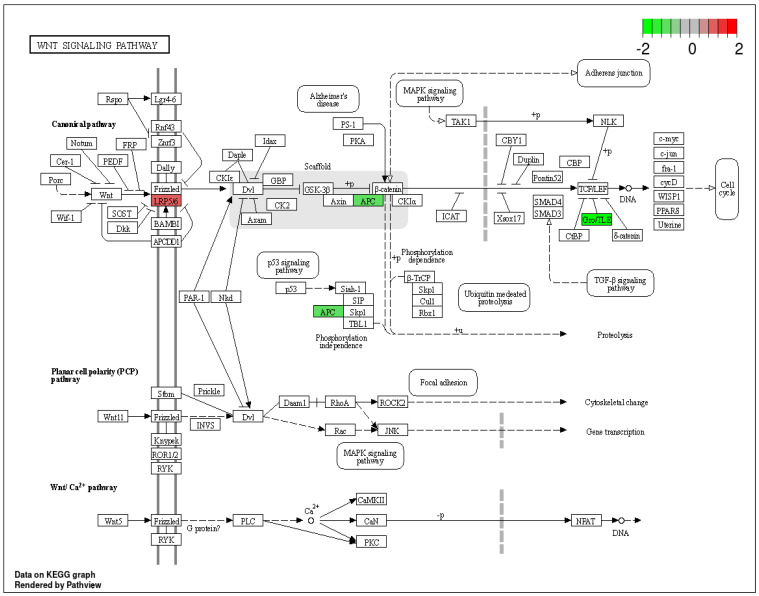
WNT signaling pathway with differentially expressed proteins (DEPs). The up-regulated DEPs are highlighted in red and the down-regulated DEPs are highlighted in green.

On the other hand, DEPs such as *LRPS*, *APC*, and *TLE* are associated with Wnt signaling pathways. These pathways are crucial for various biological processes, including embryonic development, homeostasis, cell differentiation, polarity, proliferation, and migration, primarily through the β-catenin binding of Wnt target genes. Dysregulation of Wnt signaling has been linked to multiple diseases, including cancer [27]. In head and neck squamous cell carcinoma (HNSCC), the function of the *APC* tumor suppressor protein, essential for maintaining the integrity of the β-catenin destruction complex, is frequently disrupted. This disruption often occurs due to a loss of heterozygosity and hypermethylation of the *APC* gene, leading to reduced expression in 25% to 39% of patient samples. This indicates that a subset of HNSCC cases may involve Wnt pathway activation in the carcinogenic process [28]. Therefore, the DEPs enriched in this pathway (see Figure 8) play a significant role in the mechanisms underlying HNCs.

### 2.4. Protein–Protein Interaction Network Analysis

**Figure 9:**
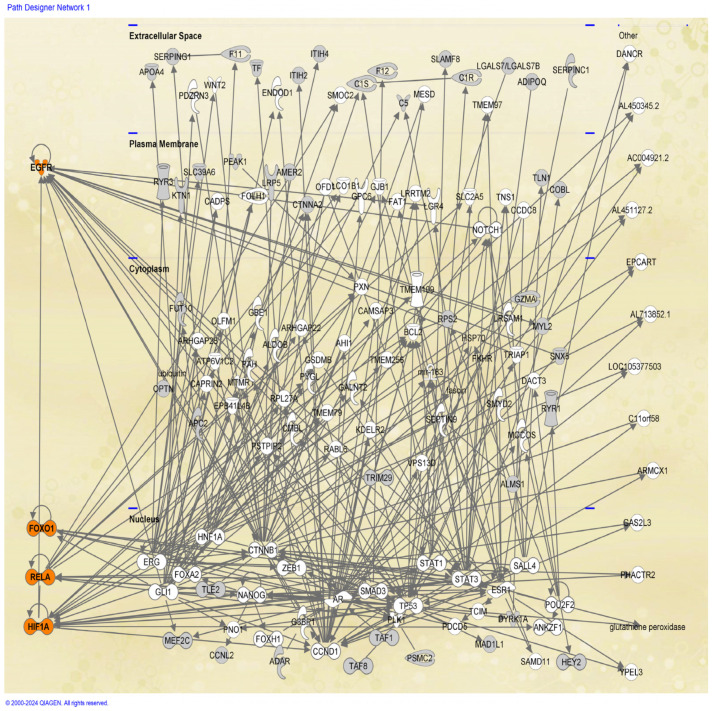
Protein–protein interaction (PPI) performed by Ingenuity pathway analysis (IPA). Four core hubs were included in the PPI network; the lead protein of each hub is shown by an orange color. The DEPs were highlighted as grey nodes.

Proteins often interact with each other to carry out their functions within cells. Therefore, we interrogated the IPA software to construct a protein–protein interaction (PPI) network. We searched for direct interactions, focusing the analysis on the DEPs identified in the study. Significantly, 47/55 proteins were associated in a single pathway related with cancer (*p*-value range: 1.54 × 10^−2^–9.16 × 10^−9^). The DEPs were represented as gray nodes in the PPI network, which included four key hubs (colored in orange) *(*Figure 9).

The first hub was the *EGFR* protein. *EGFR* interacted with 16 other proteins, including the down-regulated *MYL2* and *MEF2C*, as well as the up-regulated Sorting nexin-5 (*SNX5*) and *HIF-1α* DEPs. Overexpression of *EGFR* is associated with decreased overall survival, increased resistance to treatment, local treatment failure, and a greater likelihood of developing distant metastases in HNCs [29]. The DEPs (*MYL2*, *MEF2C*, and *SNX5*) which interacted with *EGFR* also influence the development of HNCs [30,31,32].

The second hub was the *HIF-1α* protein, which interacted with 16 other proteins. HIF-1α is an oxygen-sensitive transcription factor that plays a crucial role in various cancers including HNCs.

The third hub revolved around the *RELA* protein, which interacted with 10 other proteins. *RELA* is a transcription factor involved in HNC carcinogenesis. High levels of *RELA* have been associated with decreased sensitivity to radiation and chemotherapy in advanced HNCs [33].

The fourth hub centered on the *FOXO1* transcription factor, which interacted with five other proteins. *FOXO1* has been reported to play an important role in human cancers [34]. Its interaction with catenin beta 1 (*CTNNB1*) in the PPI network may suggest a role for FOXO1 in HNCs, as *CTNNB1* is one of the proteins known to be involved in HNCs [35]

### 2.5. DNA Damage Response Proteins

Of the DEPs, nine (16.4%) are known to play roles in the cellular response to DNA damage caused by cancer treatments. These include *TRIM29*, *PER2*, *EGFR*, *DYRK1A*, *PSMC2*, *RELA*, *CTNNA2*, *FOXO1*, and *HIF1A*, which were found to be dysregulated in our study (Table 1).

### 2.6. Predictive Biomarkers

Ten DEPs with high confidence scores were selected, the significance of their relative abundance was calculated (Table 2), and three promising predictive biomarkers, which showed statistical significance, were identified. These are *ADIPOQ*, *HEY2*, and *FUT10*. These proteins were up-regulated at the baseline of the non-responders group. Therefore, the up-regulation of these proteins at baseline were associated with an unfavorable prognosis.

**Figure 10:**
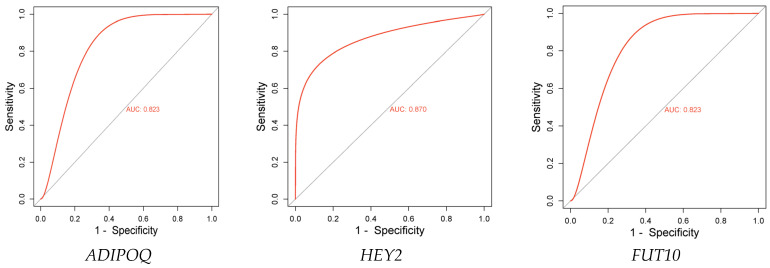
ROC curve of the DEPs. AUCs of the predictive proteins were 0.823, 0.870, and 0.823 respectively.

In addition to the significant relative abundance, the ability of these biomarkers to predict the treatment response of the individual patient was evaluated by a receiver operating characteristic (ROC) curve. The area under the ROC curve (AUC) of the three proteins, *ADIPOQ*, *HEY2*, and *FUT10*, were 0.823, 0.870, and 0.823, respectively, showing a moderate to high potential of the biomarkers to predict unfavorable treatment responses (Figure 10).

## 3. Discussion

To the best of our knowledge, this is the first study in Ethiopia to assess treatment response predictive biomarkers in HNC patients. We have identified three predictive biomarkers, *ADIPOQ*, *HEY2*, and *FUT10*, which may help to differentiate HNC patients who respond to conventional treatment. This aligns with recent findings in parotid gland tumors, where molecular diagnostic approaches have been emphasized to improve treatment strategies and patient outcomes [36]. The identification of such biomarkers not only enhances our understanding of tumor biology, but also supports the development of personalized treatment plans that could lead to better prognoses for patients.

Among 55 DEPs, eight proteins including *EGFR*, *HIF-1α*, *CTNNA2*, *RELA*, *PSMC2*, *MYL2*, *SNX5*, and *FUT2* have been previously identified as predictive biomarkers in HNCs. These proteins are involved in the inflammatory response, immune response, cellular signaling, and cellular response to hypoxic conditions.

Notably, the role of inflammation in cancer progression is further supported by studies that have demonstrated how inflammatory markers, such as neutrophil-to-lymphocyte and platelet-to-lymphocyte ratios, serve as predictors in assessing dysphagia severity and quality of life in nasopharyngeal cancer patients following RT [37]. Additionally, specific treatment parameters and patient characteristics significantly influence dysphagia severity and treatment duration, highlighting the necessity for comprehensive management strategies to enhance patient outcomes and quality of life, especially given the considerable impact of dysphagia in (HNC) patients [38]. Further research underscores how dysphagia deteriorates quality of life and emphasizes the importance of appropriate measurement tools for assessing its effects on patients’ physical, emotional, and social well-being. Integrating these insights into treatment planning can improve overall outcomes, reinforcing the need for a holistic approach to managing HNC [39,40].

The overexpression of *RELA* (NF-kB p65 subunit) is associated with a poor prognosis [41], which aligns with our findings, where *RELA* was up-regulated in the non-responder group (T/B = 0.36, *p* < 0.05). Therefore, as demonstrated in previous reports, *RELA* represents a potential therapeutic target for HNCs.

Additionally, *HIF-1α* is up-regulated in many forms of cancer and is associated with cancer pathogenesis [42,43]. However, there is a report that showed that high HIF-1α expression was associated with a better prognosis in patients with T1/T2 and node-negative squamous cell carcinoma of the tongue base treated with RT. Furthermore, head and neck tumors with high *HIF-1α* expression tend to be more sensitive to RT due to the facilitated generation of reactive oxygen species in a more vascularized microenvironment [44]. This finding is consistent with our study results, where *HIF-1α* was significantly up-regulated in the responder group at baseline (T/BL = 0.14, *p* < 0.05).

On the other hand, *EGFR* was overexpressed in many epithelial carcinomas including HNCs, which exhibited *EGFR* overexpression in up to 90% of tumors. *EGFR* plays a critical role in HNC growth, invasion, metastasis, and angiogenesis [45]. We observed that *EGFR* is up-regulated in the non-responders group (T/BL = 0.28, *p* < 0.05), consistent with previous reports where patients with higher levels of baseline *EGFR* expression had a poor outcome to treatment. *EGFR* has already been recognized as a potential target for the treatment of HNCs [32,46]. The other identified biomarkers were *PSMC2* (T/BL = 10.87), *MYL2* (T/BL = 2.01), *FUT2* (T/BL = 0.43), *CTNNA2* (T/BL = 0.35), and *SNX* (T/BL = 0.43). These proteins are involved in various activities such as protein degradation, cellular structure, cell–cell adhesion, cell–cell interaction, and cell surface expression, as well as intracellular trafficking. At baseline, *PSMC2* and *MYL2* were down-regulated, while *FUT2* was up-regulated in the responder group, which may predict a good response to treatment. In contrast, *CTNNA2* and *SNX* were up-regulated in the non-responder group, which could predict a poor response [30,31,47,48,49].

Approximately 9% of the DEPs were members of the complement and coagulation cascades. A previous study showed that combining anti C1-INH with radiation could improve radiation therapy [50]. Here, we found that *SERPING1* was significantly down-regulated in the non-responder group, which may indicate a poor response.

Generally, complement activation in the tumor microenvironment enhances tumor growth and increases metastasis [51]. However, in the context of RT, studies have reported that the production of anaphylatoxins is a crucial initial event in RT-induced immunity and clinical efficacy. Interestingly, fractionation of RT in combination with radiation holidays can help achieve an acute inflammatory response, rather than a chronic one. This acute inflammatory response appears to promote protective immunity and improve therapeutic efficacy [52]. Contradicting this, we have observed that *C5*, a component of the complement system, is significantly up-regulated (T/B = 7.39, *p* < 0.05) in the non-responder group after treatment. This suggests that the pro-tumoral effect of complement activation, in the context of chronic inflammation, may contribute to tumor resistance to RT [53], and could also be due to cumulative effect from chemoradiotherapy.

*GZMA* and *TF* were significantly up-regulated in the responder and non-responder group, respectively. *GZMA* is a type of serine protease enzyme. It is found exclusively within the granules of cytotoxic T lymphocytes (CTLs) and natural killer (NK) cells. Increased expression or up-regulation of *GZMA* has been associated with better prognosis and outcomes in cancer patients [54,55], which is in agreement with our findings. On the other hand, Serotransferrin is a serum glycoprotein in humans and plays an important role in iron metabolism. Several studies have highlighted abnormal *TF* expression in highly malignant and easily metastatic tumors [56]; this is also in-line with our study, where *TF* was up-regulated in the non-responder group.

Additionally, proteins related to ion channels in calcium signaling such as *RYR1* and *RYR3* were significantly up-regulated in the non-responder group. The ryanodine receptors are among the key ion channels in Ca^2+^ signaling, which is implicated in a variety of processes important for tumor progression. Previous studies have reported that high expression of the RYR1 isoform was associated with tumor progression in uterine serous cancer [57,58]. Using KEGG pathway analysis, we have observed that these RyR proteins were involved in several signaling pathways related to tumor biology, including the Apelin signaling pathway, oxytocin-signaling pathway, and calcium signaling pathway (see Supplementary Table S2). Given the central role of RyRs in calcium signaling, and the involvement of these proteins in pathways relevant to tumor progression, we speculate that the expression and activity of RyRs such as RYR1 may have an effect on the response of HNC patients to treatments. In other words, aberrant calcium signaling mediated by RyRs could potentially influence the efficacy of treatment in HNC patients, though further investigations would be needed to directly test this hypothesis.

On the other hand, DNA double-strand breaks (DSBs) are a severe form of DNA damage that can be induced by various genotoxic stressors, such as ionizing radiation, replication errors, and certain chemotherapies [59].

Identifying the DNA damage response caused by cancer treatment is crucial for improving patient outcomes, optimizing treatment strategies, managing side effects, and advancing cancer research. It is a key area of focus for personalized medicine and the development of new cancer therapies.

In this study, we have identified proteins that play a role in DNA damage sensing and checkpoint activation, such as *TRIM29*, *PER2*, *EGFR*, and *DYRK1A*. These proteins help recruit and activate DNA repair factors, coordinate the cellular response, and promote cell survival under genotoxic stress [60,61,62]. Other proteins that have roles in DNA repair processes, such as *PSMC2*, *RELA*, and *CTNNA2*, were identified. These proteins are known to regulate the proteasomal degradation of repair proteins, nuclear translocation of transcription factors, and cell–cell adhesion changes to facilitate DNA repair [63,64]. Transcription factors including *FOXO1* and *HIF1A* were also among the identified DNA damage response proteins, and are known to play central roles in regulating apoptosis, the cell cycle, and DNA repair in response to DNA damage. *FOXO1* orchestrates programs of gene expression that control these processes, while *HIF1A* interacts with and stabilizes key DNA repair proteins [65,66].

An analysis of the statistical significance of the relative abundance of proteins with high confidence scores revealed three candidate biomarkers, including *ADIPOQ*, *HEY2*, and *FUT10*. These proteins were overexpressed in the non-responder group, with an AUC of 0.823, 0.870, and 8.23. Briefly, the fucosyltransferase (FUT) family of proteins plays a considerable role in cancer biology. The aberrant expression of various FUT isoforms has been observed in many different types of cancer cells. Recent studies have revealed that increased FUT expression is a signature of malignant cell transformation and contributes to many abnormal events during cancer development, such as uncontrolled cell proliferation, tumor cell invasion, angiogenesis, metastasis, immune evasion, and therapy resistance [67]. Specifically, increased expression of fucosylated polysaccharides like sialyl Lewis X (SLeX) has been associated with invasion and metastasis in HNC stem cells. Indeed, various strategies for developing FUT inhibitors have been proposed in recent years, and significant advances have been observed in this area of cancer research and drug development [68]. While the role of the *FUT10* (UniProtKB-Q6P4F1) isoform in cancer is not yet well-studied, the current findings suggest that as a member of the α-1,3-fucosyltransferase family, it may play an important role not only as a prognostic marker, but also as a potential target for therapeutic interventions in HNCs. Furthermore, what determines the importance of FUT expression in tumor biology is their precise and influenced transcriptional and post-transcriptional regulation pathways, coordinated by multifactorial stimuli related to the immunological and developmental status of the tumor cell and its microenvironment [69]. Glycosylation is a critical post-translational modification that significantly affects various cellular functions [70,71]. Dysregulation of the glycosylation of cell-surface and serum glycoproteins has been associated with the development and progression of cancer [72,73]. Post-translational modifications, such as fucosylation, represent a vital regulatory mechanism that can be disrupted during cancer development, tumor progression, and metastasis [74].

Recent studies have increasingly highlighted the important and specific roles that fucosylated glycoconjugates play in tumor formation and their relationship with the recognized hallmarks of cancer [75]. Moreover, abnormal fucosylation in tumors is often linked to the activation of receptors like *EGFR* and TGF-βR, which affect the functions of integrins, selectins, and apoptotic signaling pathways, ultimately impacting tumor growth, invasion, cell death, and metastasis. Thus, fucosyltransferases (FUTs) are essential for regulating fucosylation. The abnormal expression of FUTs may serve as a potential clinical biomarker and therapeutic target to enhance survival rates in various cancers, including HNC [76].

*ADIPOQ* (UniProtKB-Q15848) is one of the most abundant hormones found in the plasma. It is a well-known regulator of glucose levels, lipid metabolism, and insulin sensitivity, exerting anti-inflammatory, anti-fibrotic, and anti-oxidant effects [77]. Many studies have reported that decreased expression of *ADIPOQ* is associated with an increased risk of various types of cancer, including colorectal, endometrial, gastric, liver, and breast cancer, among others [78]. However, a case–control study conducted by Dalamaga et al. found that an overexpression of *ADIPOQ* receptors 1 and 2 is associated with an increased risk of pancreatic cancer [79]. In line with this, we have observed a significant up-regulation of *ADIPOQ* in the non-responder group of HNC patients. Additionally, individuals with obesity or a high body mass index (BMI) tend to have lower levels of adiponectin, while those who are underweight or have a low BMI typically exhibit higher adiponectin levels [80]. One case–control study found that a lower BMI is associated with an increased risk of head and neck cancer (HNC), indicating that elevated adiponectin levels may play a role [81], which is in-line with our study. Given the heterogeneity of HNCs, further research is needed to explore the association between *ADIPOQ* and the development and progression of HNC.

The other identified biomarker was *HEY2* (UniProtKB-Q9UBP5). In general, Hairy/enhancer-of-split related with YRPW motif (Hey) transcription factors are important regulators of stem cell embryogenesis. Recent studies have shown that the Hey factors play key roles in tumor metastasis, cancer cell self-renewal and proliferation, as well as the promotion of tumor angiogenesis. Specifically, *HEY2* has been implicated in the progression of various human cancers [82]. In a study conducted by Dan-Chun Wu [83], *HEY2* expression was significantly associated with poor overall and disease-free survival in patients with hepatocellular carcinoma (HCC), and its knockdown resulted in the opposite phenotype. The findings of the current study also showed that *HEY2* overexpression is associated with poor prognosis in HNCs. KEGG enrichment analysis revealed that *HEY2* is involved in the human papillomavirus infection and Notch signaling pathways, both of which play significant roles in the development and progression of HNCs (Supplementary Table S2). The overexpression of *HEY2* observed in the current study and its association with poor prognosis suggests that *HEY2* could potentially be used to predict clinical outcomes in HNC patients, which warrants further study. Similarly, growing evidence indicates that *HEY2* is overexpressed and significantly contributes to the development of various human cancers, including osteosarcoma [84], prostate cancer [85], pancreatic ductal adenocarcinomas, and hemangiomas [86], which highlights *HEY2*’s potential as a predictive biomarker in oncology.

Despite the significant findings of this study, the relatively small sample size of HNC patients should be noted. Therefore, further research with larger cohorts is essential to validate and strengthen the statistical power of the identified predictive biomarkers for clinical application.

## 4. Materials and Methods

### 4.1. Study Population and Sample Collection

This study was conducted at the Radiotherapy Center of Tikur Anbessa Specialized Hospital, College of Health Sciences, Addis Ababa University, Ethiopia. The objective was to characterize the differential proteome of head and neck cancer (HNC) patients undergoing curative chemoradiotherapy (CRT) to identify potential predictive clinical biomarkers. Using a convenience sampling method, the study included patients who were eligible for curative CRT (excluding those receiving palliative care) and were available during the specified study period. As a result, fifteen HNC patients were recruited for the analysis. The patients consisted of nine men and six women, ranging in age from 22 to 57 years, with a median age of 39 years. The cancers were located in various areas such as the oropharynx, nasopharynx, larynx, nasal cavity, and paranasal sinuses. The histopathological types of the HNCs were as follows: four poorly differentiated and one well-differentiated tonsillar squamous cell carcinoma (SCC); four undifferentiated non-keratinized nasopharyngeal carcinomas (NPCs); two non-keratinized adenocarcinomas of the sinonasal region; one differentiated and three undifferentiated laryngeal squamous cell carcinomas. The patients’ human papilloma virus (HPV) status was unknown and none of them were smokers. The primary tumor stages were assessed using the TNM classification system, revealing the following distribution: T1 (6.6%), T2 (46.6%), T3 (13.3%), and T4 (33.3%). In terms of lymph node involvement, 46.6% of patients had N0 and N2 each, and 6.6% had N3 stage. None of the patients had diagnosed distant metastasis (all were classified as M0). All the patients in the study received induction chemotherapy with a combination of chemotherapy agents: cisplatin combined with gemcitabine, capecitabine, paclitaxel, or 5-fluorouracil. Additionally, two patients with tonsillar involvement underwent tonsillectomy, and six patients received concurrent chemoradiotherapy with cisplatin administered weekly during radiation treatment. The treatment for all patients involved a total radiotherapy dose ranging from 59.4 Gy to 70 Gy, with an average dose of 68.2 Gy. Baseline and follow-up imaging results, using CT scans, were utilized to characterize and compare the patients’ treatment response according to the Response Evaluation Criteria in Solid Tumors (RECIST) [87]. Specifically, the disappearance of all target lesions and a significant decrease in the diameter of lesions were classified as a complete response and partial response, respectively. Conversely, a significant increase in the sum of the longest diameter of the target lesions or the appearance of new lesions was classified as a non-response. The patients were then subdivided into partial responders (PR), complete responders (CR), and non-responders (NR). In this study, the responders (R) were the sum of the partial responders and complete responders. The basic demographic and clinical characteristics of the patients are presented in Table 3. The recruitment of the study participants and collection of samples were granted approval by the Institutional Review Board (IRB) of Addis Ababa University College of Health Sciences on 20 September 2023 with a protocol number 009/20/IM. In accordance with the Helsinki declarations pertaining to the utilization of human biological samples for research, written informed consent was obtained from all study participants.

Blood samples were collected immediately before the start of radiotherapy (RT) and at the end of RT. About 5 mL of blood samples were collected using BD vacutainer SST, allowed to clot for 2 h at room temperature, centrifuged at 1000× g for 10 min, aliquoted in 1 mL Eppendorf tubes, and stored at −80 °C until analysis.

### 4.2. Sample Preparation

The serum samples were thawed and subjected to pretreatment using the High-Select™ Top14 Abundant Protein Depletion Mini Spin Columns Kit (Thermo Fisher Scientific, Vacaville, CA, USA) according to the manufacturer’s protocol. This kit is designed to remove the 14 most abundant proteins present in the serum, including Albumin, IgA, IgD, IgE, IgG, IgG (light chains), IgM, Alpha-1-acid glycoprotein, Alpha-1-antitrypsin, Alpha-2-macroglobulin, apolipoprotein A1, fibrinogen, haptoglobin, and transferrin. The before and after treatment samples were analyzed separately to see changes in protein abundance between the two samples. In general, 10 µL of the sample was added to the resin slurry in the depletion spin column. The mixture in the column was incubated with gentle end-over-end mixing for 1 h at room temperature, followed by centrifugation for 2 min. The filtrate was collected in a 2 mL Eppendorf vial and lyophilized to concentrate the protein. The protein concentration was determined using a Bradford protein assay. Then, 20 µg of protein was prepared from the determined concentration.

The downstream protein analysis was performed by adding a PH buffer and denaturant solution, as well as 50 mM ammonium bicarbonate to a 20 µg protein concentration, and incubating at 60 °C for 1 h. Next, the sample was reduced with 4 mM dithiothreitol (DTT) at 95 °C for 30 min followed by alkylation with 4 mM iodoacetamide (IAA) at room temperature for 20 min in darkness (Sigma Aldrich, Merck KGaA, Darmstadt, Germany). Then, in solution, protein digestion was performed using trypsin at a 1:50 ratio *w*/*w* (Promega Corporation, Madison, WI, USA) and incubated overnight for 37 °C with gentle shaking. Samples were desalted using stage Tip C18 (Millipore, Merck KGaA, Munich, Germany) and were dried in a vacuum system. Pre-protein separation is not strictly necessary in modern proteomics workflows like shotgun proteomics. While pre-separation of whole proteins can be beneficial for specific purposes, it is not universally required. In shotgun proteomics, the entire protein mixture is digested into peptides before any separation, allowing for high-throughput analysis without the need for prior protein separation. This approach has become dominant due to its simplicity, speed, and ability to handle complex samples directly.

Although techniques like 2D-PAGE or liquid chromatography (LC) can still be useful for targeted applications—such as focusing on specific protein isoforms or complexes—they are not essential for high-throughput proteomic analysis. Modern mass spectrometers can efficiently analyze complex peptide mixtures, often making the pre-separation of intact proteins unnecessary. Additionally, 2D-PAGE can be labor-intensive, and may miss certain proteins, particularly those that are low in abundance, hydrophobic, or have extreme sizes or changes. Pre-separation can increase complexity and time requirements and may introduce sample loss.

### 4.3. LC–MS/MS Analysis

About 5 μg of peptides were resuspended in 0.1% TFA and injected into a Dionex UltiMate 3000 nano system (Thermo Fischer Scientific, Vacaville, CA, USA) coupled with an AmaZon ETD mass spectrometer (Bruker Daltonics, Bremen, Germany). Peptide samples were loaded onto a Pepmap precolumn (2 cm × 100 µm, 5 µm), followed by separation on a 25 cm Nano column (0.075 µm, Acclaim PepMap100, C18, Thermo Fischer Scientific, Vacaville, CA, USA), and at a flow rate of 300 nL/min. Multistep 360 min gradients of ACN were used. The mass spectrometer equipped with a nanoBoosterCaptiveSpray™ ESI source, (ESI Source Solutions, LLC, Woburn, MA, USA) was operated in a data-dependent acquisition mode.

### 4.4. MS Data Analysis

For MS generation, enhanced resolution and a trap ICC (ionization and collision control) value of 400,000 were used, and for MS/MS acquisition, the ICC target was increased to 100,0000. Collision-induced dissociation (CID) MS/MS fragmentation was set to fragment the ten most abundant MS peaks (Top 20). The obtained chromatograms were elaborated using Compass Data Analysis^TM^ v.4.2 (Bruker Daltonics, Bremen, Germany), and the resulting mass lists were processed using the Mascot search engine (v.2.7.0) against the UniProt human database. The search included two missed cleavages; trypsin as an enzyme, carbamidomethyl (C) as a fixed modification, and oxidation (M) as a variable modification were set in the search parameters. Mass tolerance for all identifications was generally fixed at 1.2 Da for the precursor ions and 0.6 Da for the product ions. Data were filtered using a global FDR < 5%, and the proteins with significant differences between the responders, non-responders, and their corresponding baselines were considered differentially expressed proteins.

### 4.5. Protein Identification/Quantification

Progenesis QI for proteomics v. 4.2 (Non-linear Dynamics, Newcastle, UK) was used as a label-free quantification platform. Briefly, raw data were imported, and the ion intensity maps of all runs were used for the alignment process. Only alignment scores above 60% were accepted. Peak peaking was performed using the default sensitivity and a peak width of 0.15 min and charge states of +2, +3 and +4 were set. The survey scan data was used for the quantification of peptide ions without MS/MS data. Data was normalized to all proteins and identification was achieved using Mascot. Protein abundance was calculated using the sum of all unique peptide-normalized ion abundances for that protein on each run. To indicate the statistical peptide significance, an Anova test *p*-value ≤ 0.05) and a fold-change > 1.5 were applied. Moreover, to adjust for the multiple testing problem, adjusted *p*-values, named q-values, were also provided (q-value ≤ 0.01).

### 4.6. Bioinformatics Analysis

Gene ontology (GO) and pathway enrichment analysis of all differentially expressed proteins was performed using ShinyGo v.0.77 (http://bioinformatics.sdstate.edu/go77/ (accessed on 8 July 2024)) and the Enrichr database (https://maayanlab.cloud/Enrichr/ (accessed on 8 July 2024)). Subsequently, Ingenuity pathway analysis (IPA) v.24.0.1 was utilized to generate protein–protein interaction networks. The clustering of DEPs was processed using SRplot web server (http://www.bioinformatics.com.cn/SRplot (accessed on 21 June 2024)).

### 4.7. Statistical Analysis

Statistical analysis was carried out using the IBM SPSS 21 package. Quantitative data were expressed as median (95% CI), *p* < 0.05 was considered statistically significant, and the comparison were performed by independent *t*-test. The difference in the relative abundance of proteins between R and NR was calculated. Proteins with *p* < 0.05 and fold-changes > 1.5 were considered as DEPs. SRplot data visualization and graphing were used to generate the receiver operating characteristic curves (ROC).

## 5. Conclusions

Our study revealed a significant number of DEPs, including previously identified potential biomarkers and therapeutic targets for the diagnosis and treatment of patients with HNCs. Analysis of the significance of the relative abundance of DEPs with high confidence scores revealed three potential predictive biomarkers for HNC: *ADIPOQ*, *HEY2*, and *FUT10*. Receiver Operating Characteristic (ROC) analysis confirmed the predictive ability of these biomarkers, yielding area under the curve (AUC) values of 0.823, 0.870, and 0.823, respectively. These biomarkers are predictive of a poor response to treatment in HNC patients. However, the small sample size may limit the findings, and future studies should utilize larger cohorts and orthogonal techniques to clarify the relationship between these biomarkers and HNC prognosis. The present study, conducted using cutting-edge analytical technology like nano-HPLC, provides valuable insights for the scientific community and contributes to better management strategies for this disease. Furthermore, it inspires additional investigations in this area, fostering collaboration and innovation that could lead to improved outcomes for patients. We have also identified nine proteins known to play crucial roles in cellular responses to DNA damage caused by cancer treatments. This finding necessitates further research into the molecular mechanisms underlying these proteins’ functions. Such research is crucial for significantly improving patient outcomes, optimizing treatment strategies, and managing side effects.

## Figures and Tables

**Table 1:** Up and down-regulated differentially expressed proteins (DEPs) among responder and non-responder groups with respect to their baselines.

UniProt	Gene Name	Protein Description	FC (T/BL of R & T/BL of NR)	*p*-Value	Remark
**Up-regulated**					
MD1L1_HUMAN	*MAD1L1*	Mitotic spindle assembly checkpoint protein MAD1	12.08	0.00157	*
PRS7_HUMAN	*PSMC2*	26S proteasome regulatory subunit 7	10.87	0.00418	§
TRI29_HUMAN	*TRIM29*	Tripartite motif-containing protein 29	9.10	0.00548	*
CO5_HUMAN	*C5*	Complement C5	7.39	0.01529	§
IC1_HUMAN	*SERPING1*	Plasma protease C1 inhibitor	6.92	0.02076	§
EFNB3_HUMAN	*EFNB3*	Ephrin-B3	5.11	0.02194	*
TAF8_HUMAN	*TAF8*	Transcription initiation factor TFIID subunit 8	4.89	0.02598	*
PER2_HUMAN	*PER2*	Period circadian protein homolog 2	3.95	0.02693	*
KTN1_HUMAN	*KTN1*	Kinectin	2.96	0.0273	*
FA12_HUMAN	*F12*	Coagulation factor XII	2.85	0.03558	*
FA11_HUMAN	*F11*	Coagulation factor XI	2.69	0.03937	*
EIFCL_HUMAN	*EIF3CL*	Eukaryotic translation initiation factor 3 subunit C-like protein	2.51	0.04041	*
ITIH4_HUMAN	*ITIH4*	Inter-alpha-trypsin inhibitor heavy chain H4	2.48	0.0429	*
ANT3_HUMAN	*SERPINC1*	Antithrombin-III	2.26	0.04441	*
LEG7_HUMAN	*LGALS7*	Galectin-7	2.20	0.04513	*
ALMS1_HUMAN	*ALMS1*	Alstrom syndrome protein 1	2.03	0.04515	*
CC188_HUMAN	*CCDC188*	Coiled-coil domain-containing protein 188	2.01	0.04524	*
MLRV_HUMAN	*MYL2*	Myosin regulatory light chain 2, ventricular/cardiac muscle isoform	2.01	0.04611	§
MEF2C_HUMAN	*MEF2C*	Myocyte-specific enhancer factor 2C	1.86	0.04628	*
SLAF8_HUMAN	*SLAMF8*	SLAM family member 8	1.66	0.0477	*
LRP5_HUMAN	*LRP5*	Low-density lipoprotein receptor-related protein 5	1.54	0.04808	*
OPTN_HUMAN	*OPTN*	Optineurin	1.52	0.04941	*
**Down-regulated**					
ITIH2_HUMAN	*ITIH2*	Inter-alpha-trypsin inhibitor heavy chain H2	0.58	0.001594	*
PEAK1_HUMAN	*PEAK1*	Inactive tyrosine-protein kinase PEAK1	0.57	0.001653	*
AMER2_HUMAN	*AMER2*	APC membrane recruitment protein 2	0.55	0.00191	*
TLN1_HUMAN	*TLN1*	Talin-1 OS = Homo sapiens	0.51	0.003827	*
TUTLA_HUMAN	*IGSF9*	Protein turtle homolog A	0.50	0.004195	*
APCL_HUMAN	*APC2*	Adenomatous polyposis coli protein 2	0.53	0.005011	*
COBL_HUMAN	*COBL*	Protein cordon-bleu	0.50	0.010593	*
DSRAD_HUMAN	*ADAR*	Double-stranded RNA-specific adenosine deaminase	0.48	0.011177	*
CCNL2_HUMAN	*CCNL2*	Cyclin-L2	0.48	0.012014	*
FOXO1_HUMAN	*FOXO1*	Forkhead box protein O1	0.46	0.013412	*
MYLK4_HUMAN	*MYLK4*	Myosin light chain kinase family member 4	0.44	0.014061	*
SNX5_HUMAN	*SNX5*	Sorting nexin-5	0.43	0.017811	§
FUT2_HUMAN	*FUT2*	Galactoside alpha-(1,2)-fucosyltransferase 2	0.43	0.018673	§
ADIPO_HUMAN	*ADIPOQ*	Adiponectin	0.42	0.020975	*
GRAA_HUMAN	*GZMA*	Granzyme A	0.40	0.021991	
APOA4_HUMAN	*APOA4*	Apolipoprotein A-IV	0.39	0.022091	*
RS2_HUMAN	*RPS2*	40S ribosomal protein S2	0.39	0.0244	*
FUT10_HUMAN	*FUT10*	Alpha-(1,3)-fucosyltransferase 10	0.38	0.025601	*
TF65_HUMAN	*RELA*	Transcription factor p65	0.36	0.026297	§
NEAS1_HUMAN	*NEXN-AS1*	Putative uncharacterized protein NEXN-AS1	0.35	0.027256	*
CTNA2_HUMAN	*CTNNA2*	Catenin alpha-2	0.35	0.029454	§
S39A6_HUMAN	*SLC39A6*	Zinc transporter ZIP6	0.33	0.03063	*
MRM3_HUMAN	*MRM3*	rRNA methyl transferase 3, mitochondrial	0.32	0.032547	*
TAF1_HUMAN	*TAF1*	Transcription initiation factor TFIID subunit 1	0.31	0.033653	*
TLE2_HUMAN	*TLE2*	Transducin-like enhancer protein 2	0.07	0.037239	*
RYR3_HUMAN	*RYR3*	Ryanodine receptor 3	0.30	0.040102	*
EGFR_HUMAN	*EGFR*	Epidermal growth factor receptor	0.28	0.04128	§
DYR1A_HUMAN	*DYRK1A*	Dual specificity tyrosine-phosphorylation-regulated kinase 1A	0.26	0.048222	*
CNN3_HUMAN	*CNN3*	Calponin-3	0.25	0.046347	*
TRFE_HUMAN	*TF*	Serotransferrin	0.21	0.048463	*
HIF1A_HUMAN	*HIF1A*	Hypoxia-inducible factor 1-alpha	0.14	0.048781	§
HEY2_HUMAN	*HEY2*	Hairy/enhancer-of-split related with YRPW motif protein 2	0.11	0.04896	*
RYR1_HUMAN	*RYR1*	Ryanodine receptor 1	0.05	0.049402	*

*, Proteins identified in our research; §, proteins identified in previous studies.

**Table 2:** Comparison of ten differentially expressed proteins (DEPs) with high confidence scores between Responders and Non-Responders. *, shows statistical significance.

UniProt	UniProtKB	Protein Description	R	NR	*p*-Value
ADIPOQ	Q15848	Adiponectin	0.499415509	0.002531	0.001 *
FUT10	Q6P4F1	Alpha-(1,3)-fucosyltransferase 10	0.014785713	0.060411	0.008 *
HEY2	Q9UBP5	Hairy/enhancer-of-split related with YRPW motif protein 2	0.01951626	0.319768	0.04 *
ALMS1	Q8TCU4	Alstrom syndrome protein 1	0.054899391	0.011416	0.134
TLE2	Q04725	Transducin-like enhancer protein 2	0.002374986	0.005053	0.350
RYR3	Q15413	Ryanodine receptor 3	0.007127366	0.008771	0.420
CC188	H7C350	Coiled-coil domain-containing protein 188	0.182466033	0.149482	0.461
ITIH4	Q14624	Inter-alpha-trypsin inhibitor heavy chain H4	0.162586741	0.345088	0.530
TF	P02787	Serotransferrin	0.017079943	0.021802	0.542
F12	P00748	Coagulation factor XII	0.039748057	0.075678	0.824

**Table 3:** Basic characteristics of head and neck cancer patients (N = 15).

Clinical and Demographic Features	Cases (#15)	Responders_R (N = 11)	Non-Responders_NR (N = 4)	*p*-Value
Age median (95% CI range)	39 (31.2–43.4)	39 (30.7–44.7)	33 (29.2–40.8)	
Radiation dose (95% CI)	70 (59.2–71.5)	70 (66.8–70.2)	70 (58.9–75.7)	
Gender				
Male	9 (60%)	8 (72.7%)	1 (25%)	0.141
Female	6 (40%)	3 (27.2%)	3 (75%)	
TNM-stage				
T1–T2 (T1 = 1, T2 = 7)	8 (53.3%)	7 (63.6%)	1 (25.0%)	0.506
T3–T4 (T3 = 2, T4 = 5)	7 (46.7%)	4 (36.4%)	3 (75%)	
N0	7 (53.3%)	5 (45.5%)	2 (50.0%)	0.143
N+ (N2 = 7, N3= 1)	8 (46.7%)	6 (54.5%)	2(50.0%)	
Overall stage				
I–II (I = 1; II = 2)	3 (20.0%)	2 (18.2%)	1 (25.0%)	0.69
III–IV (III = 10, IV = 2)	12 (80.0%)	9 (81.8%)	3 (75%)	

## Data Availability

All data generated or analyzed during this study were included in this published article. In addition, the primary data could be achieved from the corresponding author.

## Supplementary Materials

The following supporting information can be downloaded at: https://www.mdpi.com/article/10.3390/ijms252312513/s1.

## Abbreviations

AUC	Area Under the Curve
BD-SST	Blood collection Serum Separator Tube
BMI	Body Mass Index
BP	Biological Process
CC	Cellular Component
CRT	Chemoradiotherapy
CT	Computed Tomography
CTNLs	Circulating T-Lymphocytes
DEPS	Deferentially Expressed Proteins
DNA	Deoxyribonucleic acid
DSBs	Double Strand Breaks
FC	Fold-change
GLOBOCAN	Global Cancer Observatory
GO	Gene Ontology
GY	Gray
HNC	Head and Neck Cancers
HNSCC	Head and Neck Squamous Cell Carcinoma
HPLC	High Performance Liquid Chromatography
HPV-HR	High-risk Human Papilloma Virus
IPA	Ingenuity Pathway Analysis
MF	Molecular Function
MS	Mass Spectrometer
NK	Natural Killer cell
NPC	Nasopharyngeal Cancer
NR	None-responder
OSCC	Oral Squamous Cell Carcinoma
PCA	Principal Component Analysis
PPI	Protein Interaction
PR	Partial Responder
R	Responder
RNA	Ribonucleic acid
RECIST	Response Evaluation Criteria In Solid Tumor
ROC	Receiver operating Characteristic
RT	Radiotherapy
TFA	Trifluoroacetic acid
TNM	Tumor, Nodes, and Metastasis
